# Marginal integrity of classical and bulk-fill composite restorations in permanent and primary molars

**DOI:** 10.1038/s41598-022-18126-7

**Published:** 2022-08-11

**Authors:** Blend Hamza, Marcus Zimmerman, Thomas Attin, Tobias T. Tauböck

**Affiliations:** 1 Clinic of Orthodontics and Pediatric Dentistry, Center of Dental Medicine, University of Zurich, Plattenstrasse 11, 8032 Zurich, Switzerland; 2 Clinic of Conservative and Preventive Dentistry, Center of Dental Medicine, University of Zurich, Plattenstrasse 11, 8032 Zurich, Switzerland

**Keywords:** Dental materials, Dental treatments, Paediatric dentistry, Restorative dentistry

## Abstract

Bulk-fill composites enable timesaving and less technical-sensitive application of restorations. This study investigated and compared the marginal integrity of classical and bulk-fill composite restorations in primary and permanent molars before and after thermo-mechanical loading (TML). Two Class II cavities were prepared in each of 20 primary and 20 permanent molars. The molars were randomised in four groups for each molar type. Groups 1 and 5 were restored with a high-viscous bulk-fill composite (Tetric PowerFill), groups 2 and 6 were restored with a flowable bulk-fill composite (Tetric PowerFlow), groups 3 and 7 were restored with a high-viscous classical composite (Tetric Prime), and groups 4 and 8 were restored with a flowable classical composite (Tetric EvoFlow). In permanent molars, the flowable composites were covered with a 2-mm layer of high-viscous composite (groups 6 and 8). The restorations were subjected to TML in a custom-made chewing machine (5–50 °C, 2 min dwelling time, × 1000; 400 ,000 loading cycles, 1.7 Hz, 49 N), and quantitative marginal analysis was conducted using scanning electron microscopy. Marginal integrity of each restoration was calculated as a percentage of continuous margins before and after TML. The tested high-viscous bulk-fill restoration showed similarly high marginal integrity in primary and permanent molars as the classical restoration. The tested flowable bulk-fill restoration showed the lowest marginal integrity compared to all other restorations after TML. In contrast to flowable bulk-fill restorations, high-viscous bulk-fill restorations show similar marginal integrity as classical hybrid composite restorations after TML, in both primary and permanent molars.

## Introduction

During the last few decades, resin-based composite restorations have increasingly pushed amalgam aside as the preferable restorative material in both permanent and primary molars^1,2^. Composite restorations exhibit good mechanical and tribological properties, which lead to low annual failure rate (1.1%) in vivo^3–5^. Classically, composite restorations were inserted in approximately 2-mm-thick layers into the tooth cavity, following the so-called “incremental layering technique”. This technique is, however, regarded as time-consuming and a possible source of air entrapment between the consecutive composite layers. To overcome the aforementioned shortcomings, bulk-fill composites, which can be inserted in up to 4–6 mm layers into the cavity, were introduced^2,6^. Similar to classical composite, bulk-fill composite materials are available in low- and high-viscosity forms. While some studies documented the superiority of bulk-fill composites to classical composites with regard to lower polymerisation shrinkage stress^7–9^ and higher marginal integrity^10,11^, other studies reported the opposite^12,13^.

The fact that bulk-fill composite materials can be inserted in large material volumes raised the concern about the resulting polymerisation shrinkage stress at the tooth-restoration interface. In addition to this volumetric shrinkage, another contributing factor to shrinkage stress is the viscoelastic behaviour of the material during polymerisation. To reduce interfacial shrinkage stresses, prepolymer stress relievers and stress-relaxant polymerization modulators have been integrated in bulk-fill composite materials. Applying an intermediate layer of low-modulus flowable composite material on the cavity walls has also been shown to reduce the shrinkage stress^7,14^.

It is logical to assume that the timesaving benefit, offered by bulk-fill composites, is even more advantageous when treating children compared to adults. Shorter treatment times might help achieve a better patient compliance^15,16^. The differences in the enamel structure between primary and permanent teeth (lower calcium and phosphate concentration, thinner thickness and occlusal direction of the cervical enamel rods for primary teeth) also lead to different behaviour when treated with adhesive systems^17,18^. Nevertheless, the performance of bulk-fill restorations in terms of marginal integrity and how this behaves on primary molars, in comparison to permanent molars, has not yet been investigated.

This in-vitro study was therefore carried out to investigate and compare the marginal integrity of classical and bulk-fill composite restorations in primary and permanent molars before and after artificial aging. The null-hypotheses of the study were that (1) there are no differences in the marginal integrity of the tested composite materials between primary and permanent teeth, and (2) there are no differences in the marginal integrity between the tested restorations in primary and permanent teeth.

## Materials and methods

Twenty sound primary and twenty sound permanent human molars were used in this in-vitro study. Molars were extracted due to periodontitis or orthodontic reasons and were stored in 0.1% thymol solution at 4 °C until use. All patients and/or legal guardians gave their written informed consent for the use of their teeth for research purposes and all molars were irreversibly anonymised immediately after extraction. The study was therefore carried out in accordance with the Federal Act on Research involving Human Beings (Human Research Act; article 2, paragraph 2) and the authorisation from the ethics committee was waived (Zurich cantonal ethics commission, BASEC-2021-00,635). To facilitate their handling, the roots of all molars were embedded in acrylic resin (Paladur, Heraeus Kulzer, Hanau, Germany) and mounted on custom-made holders. Two standardised Class II cavities were prepared mesially and distally in each molar (mesio-occlusal and disto-occlusal cavity). The proximal cavities in primary molars were 4 mm in width, 2 mm in axial depth and 3 mm in occlusal-gingival depth. The proximal cavities in permanent molars were 5 mm in width, 3 mm in axial depth and 4 mm in occlusal-gingival depth. The width, axial depth and mesio-distal depth of the occlusal cavities were kept at 2 mm in both primary and permanent molars. Cavity preparation was carried out using 80-µm cylindrical diamond burs (Universal Prep Set, Intensiv, Grancia, Switzerland) mounted on a high-speed contra-angle handpiece (Sirius, Micro-Mega, Besançon Cedex, France) rotating at 40,000 rpm. A new bur was used after the preparation of four cavities. All cavity margins ended within enamel. Any unsupported enamel was chiseled away and enamel margins were not further beveled. The prepared molars were then randomised into eight groups (n = 10 per group; groups 1–4: primary molars, groups 5–8: permanent molars) using a computer-generated randomisation table (Microsoft Excel). A recently conducted study was used as an orientation for the here used sample size^11^.

**Figure 1:**
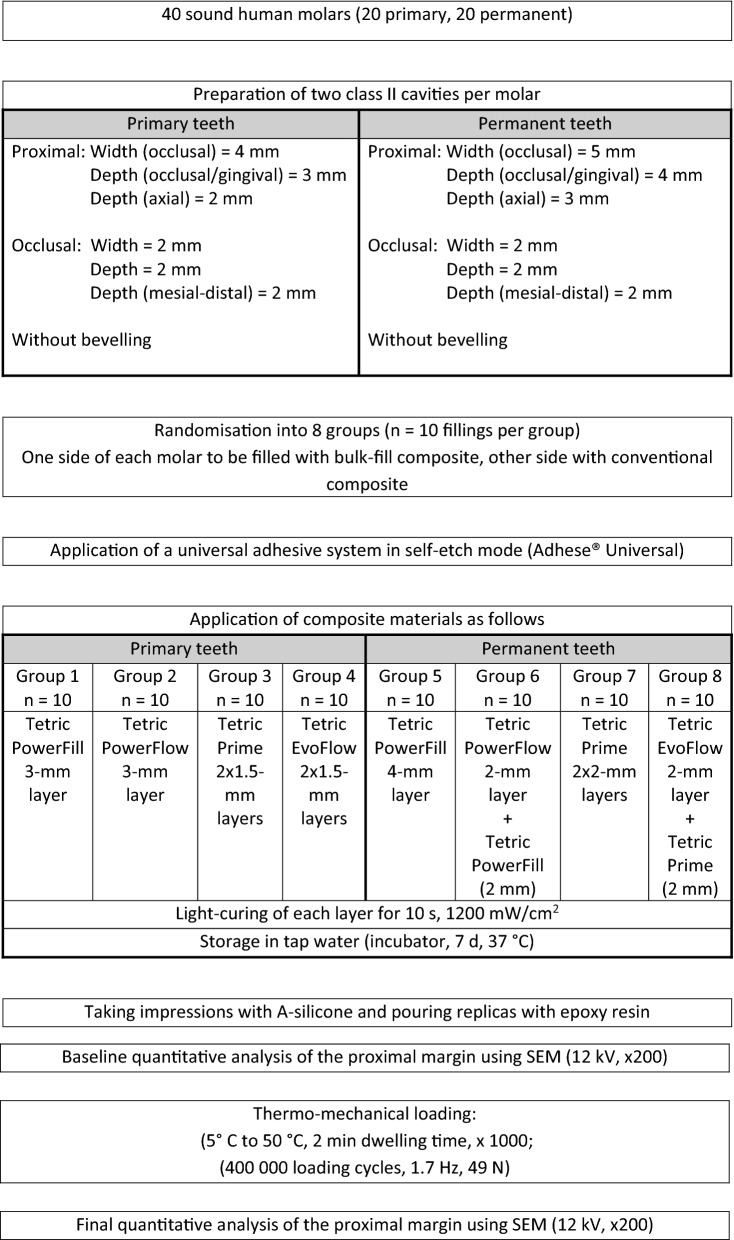
Study design.

For restoration, each molar was mounted on a custom-made adjacent-tooth simulator (ZPZ laboratory, Center for Dental Medicine, Zurich, Switzerland). A stainless-steel matrix band (Omni-Matrix sectional, regular, Ultradent Products, South Jordan, UT, USA) was adapted on each cavity in conjunction with a wooden wedge, which was placed 1 mm below the gingival margin. A universal adhesive was applied on enamel and dentine in self-etch mode and scrubbed for 20 s (Adhese Universal, Ivoclar Vivadent, Schaan, Liechtenstein). The adhesive was thinned, and the solvent was evaporated with a gentle blow of air. Thereafter, the adhesive was light-cured for 10 s at 1200 mW/cm^2^ (Bluephase G4, Ivoclar Vivadent). The cavities were then restored based on their experimental group as shown in Fig. 1. Two bulk-fill composite materials (Tetric PowerFill “high viscous” and Tetric PowerFlow “flowable”) and two classical composite materials (Tetric Prime “high viscous” and Tetric EvoFlow “flowable”) were used to restore the molars. The composition of the tested composite materials is shown in Table 1. When permanent molars were to be restored with flowable composites (groups 6 and 8), a 2-mm layer of the respective high-viscous composite (classical or bulk-fill) was applied over the polymerised flowable composite layer. Each composite layer was light-cured for 10 s at 1200 mW/cm^2^ (Bluephase G4, Ivoclar Vivadent) according to the manufacturer’s instructions. Care was taken to always place the tip of the polymerisation light as near as possible to the surface to be polymerised. All molars received one bulk-fill composite filling on one proximal side and a classical composite filling on the other side randomly for the mesial and distal surface. The proximal fillings were finished and polished using Sof-Lex discs with decreasing grit sizes (Sof-Lex Pop-on, 3M ESPE, St. Paul, MN, USA) under constant water-cooling. The occlusal parts of the fillings were polished using the OptraGloss system (HP flame, Ivoclar Vivadent). Polishing was carried out under a stereo microscope (× 4) and the Sof-Lex discs were replaced after polishing four proximal fillings. After completing the polishing, each filling was given a code on its respective side of the acrylic holder (BH). The restored molars were then stored in an incubator at 37 °C in tap water in the dark for 7 d.

**Table 1:** The tested composite materials and their compositions according to the manufacturer.

Composite materials	Monomers (wt%)	Filler (wt%)
Tetric PowerFill (high viscous bulk-fill)	Bis GMA, UDMA, Bis-EMA, Bis-PMA, DCP (17–18%)	Ba-Al-Silicate glass, copolymer, ytterbium trifluoride, mixed oxide (79–80%)
Tetric PowerFlow (flowable bulk-fill)	Bis GMA, Bis-EMA, DCP, (28–29%)	Ba-Al-Silicate glass, copolymer, ytterbium trifluoride (71–72%)
Tetric Prime (high viscous classic)	Bis GMA, UDMA, Bis-EMA (19–20%)	Ba-Al-Silicate glass, copolymer, mixed oxide, ytterbium trifluoride (79–80%)
Tetric EvoFlow (flowable classic)	Bis GMA, UDMA, D3MA (35–40%)	Ba-Al-Silicate glass, copolymer, mixed oxide, ytterbium trifluoride, silicone dioxide (60–65%)

*Bis GMA* bisphenol A-diglycidyl dimethacrylate, *UDMA* urethane dimethacrylate, *Bis-EMA* ethoxylated bisphenol A dimethacrylate, *Bis-PMA* propoxylated bisphenol A dimethacrylate, *DCP* tricyclodecane-dimethanol dimethacrylate, *D3MA* Dicandiol dimethacrylate.

At the end of the 7-d storage time, A-silicon impressions were taken for each proximal filling (President Light Body, Coltène Whaledent, Altstätten, Switzerland). The impressions were poured out with epoxy resin (Epoxyharz L, R&G Faserverbundwerkstoffe, Waldenbuch, Germany) and fixed on aluminium holders (Cementit universal, Merz&Benteli, Niederwangen, Switzerland). The replicas were sputter-coated with gold (Sputter SCD 030, Balzers Union, Balzers, Liechtenstein) and quantitatively analysed for marginal integrity (baseline analysis) using scanning electron microscopy (SEM) at 20 kV and 200 × magnification (Amray 1810/T, Amray, Bedford, MA, USA). Marginal integrity was expressed as a percentage of the assessable continuous margins for each proximal restoration^19,20^. In groups where flowable composite restorations were covered with a layer of high-viscous composite, the whole proximal margin (including flowable and high-viscous composite) was analysed. This and the final margin analysis were conducted by a calibrated and experienced investigator (MZ) who was blinded to the groups and only had access to SEM images of each filling (file names = code set for each filling by BH).

After the baseline margin analysis, all fillings were subjected to simultaneous thermo- mechanical loading (TML) in a computer-controlled masticator (CoCoM 2, ZPZ, Zurich, Switzerland). The mesial and distal filling of each molar was mechanically loaded on the occlusal part (perpendicular on the mesial and distal groove) using two Empress CAD antagonists (cusp-shaped, one for each filling) for 400,000 loading cycles at 1.7 Hz and 49 N. The bath temperature inside the masticator was changed from 5 to 50 °C with 2 min dwelling time for 1000 times^11^.

**Figure 2:**
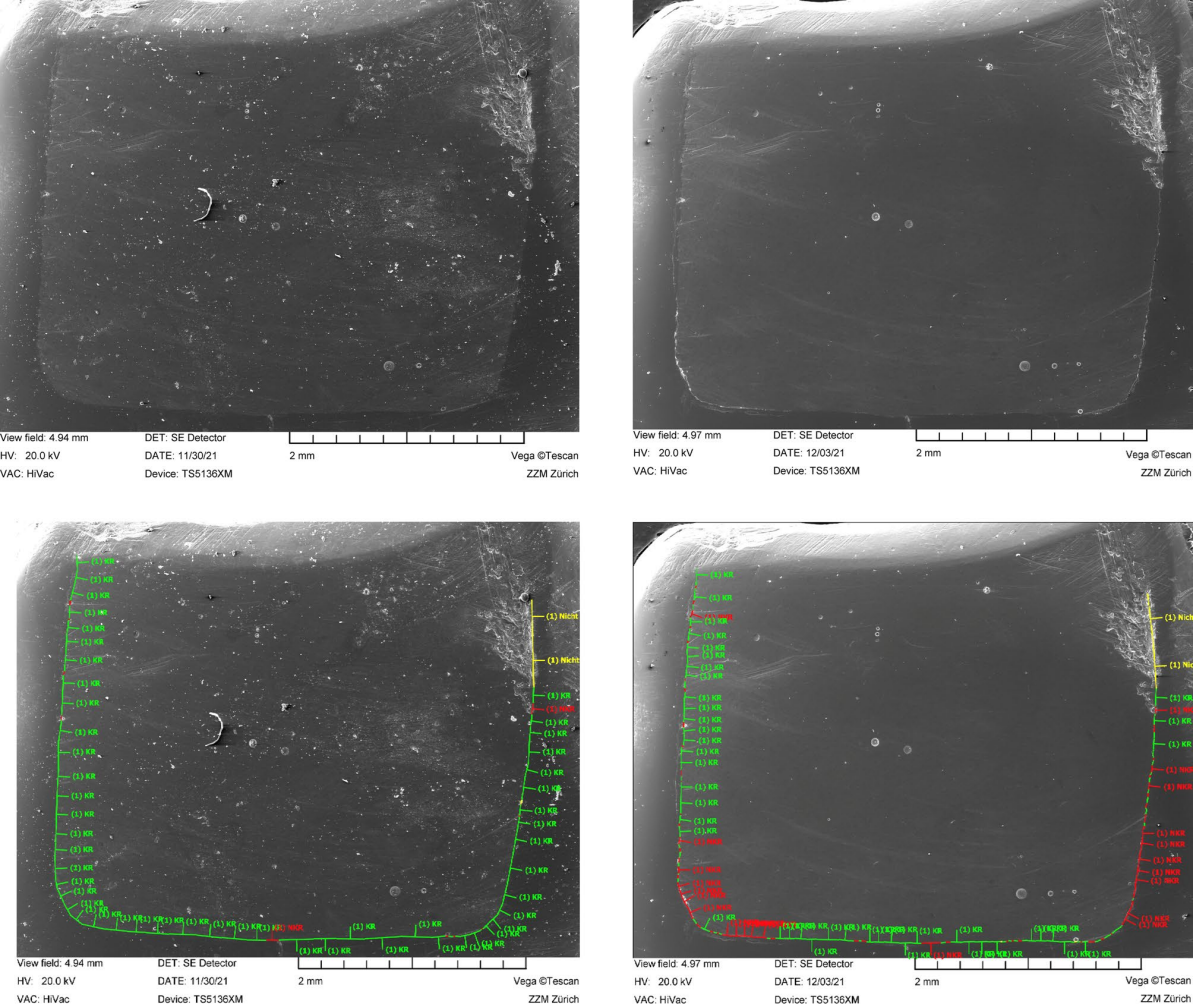
SEM images (× 200) and illustration of the quantitative marginal analysis (below) for a restoration (group 1) before (left) and after thermo-mechanical loading (right). The green line indicates continuous margin segments, the red line non-continuous margin segments, and the yellow line non-assessable margin segments.

After TML, A-silicon impressions were re-taken, and the fillings were subjected again to quantitative margin analysis (final analysis) using the same aforementioned protocol. Figure 1 summarises the study design and Fig. 2 depicts the marginal analysis of one restoration.

### Statistical analysis

A mixed effect linear model was adopted to analyse the data. The marginal integrity was set as a target variable. The restoration material, the observation time (before vs. after TMC) and the tooth type (primary vs. permanent) were set as fixed variables and the restoration ID as a random intercept. The residual analysis did not show violations of the model assumption. Pairwise contrasts were estimated and corrected for multiple testing according to Tukey. The significance level was set at α = 0.05. Data were analysed using the statistical program R (The R Foundation for Statistical Computing; Vienna, Austria; www.R-project.org).

## Results

**Figure 3:**
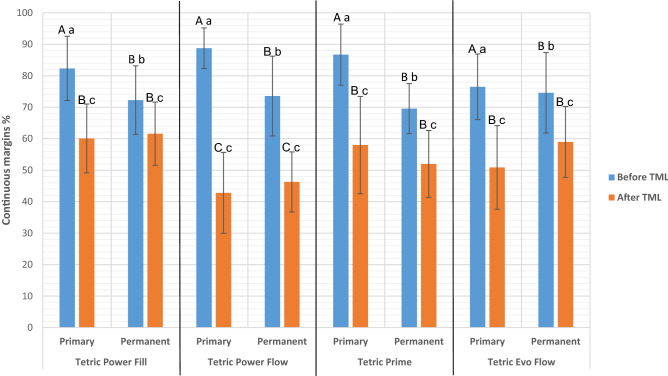
Mean and standard deviation of the percentage of continuous margins before and after thermo-mechanical loading (TML) for each experimental group. Same lower-case letters indicate no statistically significant difference within the same restoration material. Same capital letters indicate no statistically significant difference across all restoration materials (within the same molar type [primary or permanent] and the same observation time [before TML or after TML]).

Figure 3 depicts the percentage of the continuous margins (marginal integrity) for each experimental group.

*Before TML*: In primary molars, marginal integrity achieved by Tetric PowerFlow (88.8 ± 6.4%), Tetric Prime (86.7 ± 9.6%), Tetric PowerFill (82.4 ± 10.2%) and Tetric Evo Flow (76.5 ± 10.4%) were not statistically significantly different (*p* > 0.1). In permanent molars, marginal integrity achieved by Tetric EvoFlow (74.6 ± 12.7%), Tetric PowerFlow (73.6 ± 12.6%), Tetric PowerFill (72.3 ± 10.8%) and Tetric Prime (69.6 ± 7.9%), were also not statistically significantly different (*p* > 0.1). The marginal integrity in primary molars was always statistically significantly higher than that in permanent molars (*p* < 0.0001), regardless of the applied restoration.

*After TML*: All restorations showed a statistically significant reduction in their marginal integrity after TML. In primary molars, the achieved marginal integrity was as follows: Tetric PowerFill (60.1 ± 10.9%), Tetric Prime (58.0 ± 15.4%), Tetric EvoFlow (50.9 ± 13.2%) and Tetric PowerFlow (42.8 ± 12.8%). In permanent molars, the achieved marginal integrity was as follows: Tetric PowerFill (61.6 ± 10.0%), Tetric EvoFlow (59.0 ± 11.2%), Tetric Prime (52.0 ± 10.6%) and Tetric EvoFlow (46.7 ± 9.5%). In both tooth types (primary and permanent), restorations with Tetric PowerFlow showed statistically significantly lower marginal integrity compared to all other material groups (*p* < 0.05), which were not statistically significantly different from each other (*p* > 0.1).

## Discussion

Bulk-fill restorations offer timesaving benefits, which enhance efficiency, and are especially advantageous when treating children. The marginal integrity of such restorations in primary molars, in comparison to permanent molars, still needed to be comprehensively investigated. The present study shows that the tested composite materials achieved similar marginal integrity in primary and permanent teeth after TML (the first null hypothesis cannot be rejected) and that only the investigated high-viscous bulk-fill restoration achieved similar marginal integrity compared to classical composite restorations in primary and permanent teeth (the second null hypothesis has to be rejected).

To the authors’ best knowledge, this is the first study to compare the marginal integrity of composite restorations in both primary and permanent molars in the same experimental setting. The cavity dimensions, especially in primary molars, were chosen to allow the application of two layers of the classical composites or one layer of the bulk-fill composites without the risk of exposing the pulp (which would clinically indicate the application of a stainless-steel crown in primary molars^1,11^). In permanent molars, flowable composite materials were covered with a layer of high-viscous composite to overcome the reduced mechanical properties of flowable composites, as recommended in earlier studies^21–23^. In all molars, enamel margins were not beveled, and a universal adhesive was applied in self-etch mode. This is also part of the whole less-steps timesaving-restoration concept. Nevertheless, it might be assumed that better marginal integrity would have been achieved in this study, had enamel been etched with phosphoric acid and/or had enamel margins been beveled^24,25^.

TML challenges the tooth-restoration interface and can induce the formation of marginal gaps or the progression of initially existing gaps due to the different coefficients of thermal expansion of dentine/enamel and the restoration material^26^. This gap formation (i.e., lack of marginal integrity) has been associated with higher risk of marginal discoloration and/or the development of secondary caries. However, this association has not yet been clinically confirmed and the development of secondary caries was reported to rather depend on patient-related factors^27–29^. Therefore, even if marginal integrity has been reported to be able to predict the clinical performance of a restoration to some extent, it should not be used as the only criteria to make such assumptions^30^.

Before TML, all groups showed higher marginal integrity in primary molars than in permanent molars. This could be attributed to the fact that the cavities in primary molars were 1 mm less deep and less wide than those in permanent molars. In case of high-viscous bulk-fill restorations, it might be assumed that a 3-mm layer (in primary molars) would produce less shrinkage stress than a 4-mm layer (in permanent molars), resulting in lower initial marginal gap formation^31^. In case of classical restorations, however, one might argue whether the small 0.5 mm difference in layer thickness between primary (1.5-mm layers) and permanent (2-mm layers) molars could be responsible for such finding. Here, the fact that the polymerisation light was closer to the restoration material might—better—explain the finding of superior marginal integrity. Another finding of the present study was that before TML, bulk-fill and classical restorations showed similarly high marginal integrity in each molar type. This was also previously reported for primary teeth by Paganini et al.^11^. Furthermore, no differences between the tested flowable bulk-fill composite and the high-viscous bulk-fill composite was noticed in terms of marginal integrity before TML. A similar finding in terms of interfacial gap was also reported in a previous study without mechanical loading of the tested bulk-fill composite materials^32^.

After TML, all experimental groups showed a reduction in their marginal integrity. The higher marginal integrity observed in primary molars before TML could not be preserved after TML. However, all groups still showed similarly high marginal integrity in primary and permanent molars. The tested high-viscous bulk-fill composite (Tetric PowerFill) offered similarly high marginal integrity as the classical restorations in primary and permanent molars. This performance of high-viscous bulk-fill restorations is well established and reported in previous studies with permanent teeth^33^. On the other hand, the tested flowable bulk-fill composite (Tetric PowerFlow) showed the lowest marginal integrity in primary and permanent molars. This could be explained by the inferior mechanical properties of this type of composites (e.g., lower surface hardness and lower modulus of elasticity)^34^. This finding in primary molars was also reported in an earlier study, where the placed flowable bulk-fill restorations also showed lower marginal integrity in comparison to high-viscous classical and bulk-fill restorations after TML^11^. The same finding was also reported in other studies in permanent molars^35,36^. Contrary findings, which reported similar marginal integrity of flowable bulk-fill composite restorations as classical and/or high-viscous bulk-fill restorations were, however, also reported^34,37^. Different cavity preparations, TML protocols and material properties could be responsible for these different observations.

Based on the results of this in-vitro study and within its limits, it can be concluded that the tested high-viscous bulk-fill composite shows comparable marginal integrity as classical composite materials in both primary and permanent molars, before and after thermo-mechanical loading. This does not apply to the flowable bulk-fill composite under investigation, which shows similarly high marginal integrity as the other composite materials only before thermo-mechanical loading. After TML, all restorations placed in primary teeth achieved similar marginal integrity as in permanent teeth, which adds to their suitability in paediatric dentistry.

## Data Availability

The datasets generated during and/or analysed during the current study are available from the corresponding author on reasonable request.
